# *l*-tetrahydropalmatine reduces nicotine self-administration and reinstatement in rats

**DOI:** 10.1186/s40360-016-0093-6

**Published:** 2016-11-07

**Authors:** Shamia L. Faison, Charles W. Schindler, Steven R. Goldberg, Jia Bei Wang

**Affiliations:** 1 Department of Pharmaceutical Sciences, School of Pharmacy, University of Maryland, Baltimore, MD USA; 2 Preclinical Pharmacology Section, Behavioral Neuroscience Research Branch, National Institute on Drug Abuse, National Institutes of Health, DHHS, Baltimore, MD USA

**Keywords:** *levo*-Tetrahydropalmatine, Nicotine, Addiction

## Abstract

**Background:**

The negative consequences of nicotine use are well known and documented, however, abstaining from nicotine use and achieving abstinence poses a major challenge for the majority of nicotine users trying to quit. *l*-Tetrahydropalmatine (*l*-THP), a compound extracted from the Chinese herb *Corydalis*, displayed utility in the treatment of cocaine and heroin addiction via reduction of drug-intake and relapse. The present study examined the effects of *l*-THP on abuse-related effects of nicotine.

**Methods:**

Self-administration and reinstatement testing was conducted. Rats trained to self-administer nicotine (0.03 mg/kg/injection) under a fixed-ratio 5 schedule (FR5) of reinforcement were pretreated with *l*-THP (3 or 5 mg/kg), varenicline (1 mg/kg), bupropion (40 mg/kg), or saline before daily 2-h sessions. Locomotor, food, and microdialysis assays were also conducted in separate rats.

**Results:**

*l*-THP significantly reduced nicotine self-administration (SA). *l*-THP’s effect was more pronounced than the effect of varenicline and similar to the effect of bupropion. In reinstatement testing, animals were pretreated with the same compounds, challenged with nicotine (0.3 mg/kg, s.c.), and reintroduced to pre-extinction conditions. *l*-THP blocked reinstatement of nicotine seeking more effectively than either varenicline or bupropion. Locomotor data revealed that therapeutic doses of *l*-THP had no inhibitory effects on ambulatory ability and that *l*-THP (3 and 5 mg/kg) significantly blocked nicotine induced hyperactivity when administered before nicotine. In in-vivo microdialysis experiments, *l*-THP, varenicline, and bupropion alone elevated extracellular dopamine (DA) levels in the nucleus accumbens shell (nAcb).

**Conclusions:**

Since *l*-THP reduces nicotine taking and blocks relapse it could be a useful alternative to varenicline and bupropion as a treatment for nicotine addiction.

**Electronic supplementary material:**

The online version of this article (doi:10.1186/s40360-016-0093-6) contains supplementary material, which is available to authorized users.

## Background

Nicotine addiction via tobacco smoking can lead to cancer and is a leading cause of preventable, premature death [2, 1]. This correlation is well known and documented, however, according to the Center for Disease Control and Prevention there are currently 42.1 million smokers in the U.S. [5]. A large majority of these users (70 %) have the desire to quit smoking [2], but an inability to do so. Nicotine elicits its effect on users by binding to nicotinic acetylcholine receptors (nAChRs), which are found in the central as well as the peripheral nervous system [2, 7, 13]. nAChRs located in the ventral tegmental area (VTA) and nAcb modulate the release of DA in the mesolimbic DA system, triggering the reward and reinforcement of nicotine use [2, 12, 23, 24]. This reward incites the nicotine addiction cycle that can be highly difficult to break.

A variety of treatments are currently on the market to treat nicotine addiction including bupropion, varenicline, and nicotine replacement therapy [2, 22]. Bupropion acts by blocking the reuptake of DA/norepinephrine (NE) from the synapse alleviating the hypodopaminergic state associated with craving. Varenicline acts by binding nAChRs as a partial agonist, stimulating modest release of DA to alleviate the hypodopaminergic state [4, 6, 9]. Varenicline also acts as a competitive antagonist when given in the presence of nicotine. Both varenicline and bupropion, however, carry black box warnings for neuropsychiatric symptoms and suicidality [2]. Nicotine replacement therapy administers low concentrations of nicotine transdermally or via GI absorption relieving the hypodopaminergic state without creating the addictive euphoria of smoking a cigarette [2]. Despite these interventions, the effectiveness of smoking cessation in clinical trials with these treatments only reaches a success rate of 5 to 35 % [2]. Thus, the current treatments for nicotine addiction have limited effectiveness and side-effects that make the continued research and development of new anti-smoking therapeutics a necessity.

*l-*THP, is a purified compound from the Chinese plant *Corydalis*, and has been used safely for decades in China to treat chronic pain as a non-opioid analgesic [17, 19, 20, 30, 34]. *l*-THP has a favorable safety profile, as common adverse events seen in its clinical use are sleepiness, dizziness, and nausea [30]. The therapeutic potential of this herbal compound has been explored to include anti-addiction treatments for cocaine, methamphetamine, and heroin [19, 20, 28, 30, 34]. In the preclinical setting, *l-*THP has been shown to effectively reduce cocaine SA and methamphetamine reward as assessed by condition place preference testing [19, 20, 28, 31]. This efficacy is translated to the clinical setting as *l-*THP has been shown to reduce craving and relapse to heroin-seeking when given after detoxification [33]. The mechanism of action of *l-*THP is not completely known. However, it is known that *l-*THP binds to D_1_, D_2_, D_3_, serotonin (5-HT), and alpha-1 adrenergic receptors antagonistically, while binding to alpha-2 adrenergic receptors as an agonist [17, 30]. This broad binding profile of *l-*THP is believed to underlie its utility in the treatment of addiction to various classes of drugs. The utility seen in previous studies of *l-*THP as an aid in the treatment of psychostimulant and opioid addiction warrants further study of the efficacy of *l-*THP on the abuse-related effects of other drugs within these classes.

In the current study, we examined the efficacy of *l-*THP in the treatment of various behavioral models of nicotine addiction as assessed by nicotine SA, reinstatement, and locomotor hyperactivity in rats. The efficacy of *l-*THP in the treatment of nicotine SA was systematically compared with the efficacy of FDA-approved anti-smoking medications, bupropion and varenicline. This was done with the aim of making a case for clinical study of *l-*THP in the treatment of nicotine addiction. As a separate analysis, we also conducted *in vivo* microdialysis experiments that studied the effect of *l-*THP administration on extracellular DA concentrations within the nucleus accumbens shell; this effect of *l-*THP was compared to the effects of nicotine, as well as combinations of *l-*THP and nicotine, bupropion and nicotine, and varenicline and nicotine. This is the first report to provide behavioral and neurochemical evidence of the effects of *l-*THP in nicotine addiction.

## Methods

### Compounds

Nicotine hydrogen tartrate salt (Sigma Aldrich, St. Louis, MO) was dissolved in 0.9 % NaCl (Hospira, Inc, Lake Forest, IL) with pH adjusted to 7 using sodium hydroxide. *l-*THP (base), was acquired from Wuxi Gorunje Technology Co., LTD and was dissolved in 2 % tween 80 (Sigma Aldrich, St. Louis, MO), 3 % ethanol (Sigma Aldrich, St. Louis, MO), and 95 % sterile water (Hospira, Inc, Lake Forest, IL). The reported purity of *l-*THP was assessed by high performance liquid chromatography where the purity was determined as 98.85 %. Varenicline tartrate was supplied by LKT Laboratories, Inc and dissolved in 0.9 % NaCl with pH adjusted to 7 using sodium hydroxide. Bupropion was supplied by Enzo Life Sciences and dissolved in 0.9 % NaCl. 3-Hydroxytyramine (3,4 Dihydroxyphenethylamine; Dopamine Hydrochloride) (Sigma Aldrich, St. Louis, MO) was diluted to 10^−8^ with Perchloric Acid 70 % (Sigma Aldrich, St. Louis, MO) and sterile water (Hospira, Inc, Lake Forest, IL). Ketamine HCl was supplied through NIDA pharmacy. Xylazine (Sigma Aldrich, St. Louis, MO) was dissolved in 0.9 % NaCl. Equithesin (Pentobarbital Na, Chloral hydrate and Magnesium Sulfate) was supplied by NIDA. Formalin Solution 10 % was acquired from Sigma Aldrich, St. Louis, MO.

### Animals

For the behavioral experiments, male Sprague-Dawley rats (Charles River) weighing 300–325 g at the beginning of the study were individually housed and maintained in temperature- and humidity-controlled facilities fully accredited by AAALAC. Animals were housed on a 12 h/12 h dark/light cycle (lights out from 8:00 am to 8:00 pm). Behavioral experiments were conducted in the dark phase. For the microdialysis experiments, male Sprague-Dawley rats (Charles River) weighing 200–275 g at the beginning of the study were housed two to a cage and maintained in temperature- and humidity-controlled facilities fully accredited by AAALAC. Animals were housed on a 12 h/12 h light/dark cycle (lights out from 8:00 pm to 8:00 am). Microdialysis experiments were conducted in the light phase. All experimental procedures were approved and conducted in accordance with guidelines of the Institutional Animal Care and Use Committee of the Intramural Research Program National Institute on Drug Abuse, National Institutes of Health, Department of Health and Human Services and the University of Maryland, Baltimore. All treatment groups were randomly assigned for each study. Only animals meeting the specified criteria per experiment were analyzed.

### Nicotine SA and reinstatement

#### Catheterization surgery

Jugular vein catheter implantations were performed as described previously [27]. Briefly, rats were anesthetized under ketamine/xylazine (100 mg/kg, 10 mg/kg), and implanted with a catheter in the jugular vein and a mesh-based backmount just below the shoulder blades. Rats were allowed to recuperate for at least five days.

#### Nicotine SA

After the recuperation period, rats began SA training (Coulbourn Instruments, Whitehall, PA). Rats were placed on a restricted diet of chow per day (~30 g) to maintain their current weight. During 2-h training sessions, nose pokes to the correct hole resulted in an infusion (Harvard Apparatus, Holliston, MA) of 0.03 mg/kg nicotine, followed by a 20-s timeout period in which house lights flashed on and off. Training began under a fixed-ratio (FR) 1 schedule of reinforcement. Once a rat received at least 10 reinforcers for three consecutive sessions, the response criteria was increased incrementally to FR 2, FR 3, and finally FR 5. Once a rat responded at or above 10 infusions for five consecutive sessions, pretreatment with saline (i.p. 30 min), *l-*THP (3 mg/kg, 5 mg/kg i.p. 30 min), varenicline (1 mg/kg i.p. 2-h), or bupropion (40 mg/kg i.p. 30 min) began. Repeated testing consisted of three consecutive days during which rats were pretreated with one of the aforementioned drugs and allowed to self-administer nicotine during 2-h sessions. Separate rats were used for dosing groups.

#### Nicotine extinction

Extinction occurred in the same chambers as SA by removing nicotine-associated cues and replacing saline infusions for nicotine. Thus the infusion and 20-s timeout did not occur after the FR 5 criterion was met. Rats were trained under extinction criteria for at least five sessions or until responding was at or below 25 % of nicotine baseline responding. Once this criterion was met, reinstatement testing began.

#### Nicotine reinstatement

Rats were pretreated with saline (i.p.), *l-*THP (3 mg/kg, 5 mg/kg i.p.), varenicline (1 mg/kg i.p.), or bupropion (40 mg/kg i.p.) 30 min before placement into chambers and received nicotine (0.3 mg/kg s.c.) 5 min before placement into chambers. Environmental cues present during nicotine SA (20 s time out, infusion of pump) were reintroduced. During reinstatement sessions nicotine was not available, infusions of 0.03 ml/kg saline were delivered after the FR 5 criterion was met.

#### Nicotine-induced hyperactivity testing

Nicotine-induced hyperactivity testing was conducted over the course of 14 one-hr sessions. Rats were allowed to acclimate to the chambers for two sessions. Baseline readings with injections of saline 30 min and 5 min before access to locomotor chambers were taken for four sessions. On the seventh session, pretreatment with either *l-*THP (3 mg/kg, 5 mg/kg i.p.) or saline (i.p.) was given 30 min before placement into the chamber followed by an injection of 1 ml/kg saline (s.c.) 5 min before the start of the session. During sessions 8–13 rats were pretreated with *l-*THP or saline 30 min before the start of the session, followed by and injection of 0.4 mg/kg nicotine (s.c.) 5 min before the start of the session. In session 14 (Nic Challenge) rats received only a challenge dose of 0.4 mg/kg nicotine five minutes before the start of the session.

#### l-THP locomotor control

Rats were allowed two sessions to acclimate to locomotor chambers (Med Associates Inc, Georgia, VT). After the acclimation period, rats were pretreated with saline or *l-*THP (3, 4, 5 mg/kg i.p.) 30 min before locomotor sessions began. Rats received an injection of 1 ml/kg saline (s.c.) 5 min before placement into locomotor chambers. This delivery method of treatments was done to mirror injections given during hyperactivity testing. Rats were then allowed to move freely in locomotor chambers for 1-h sessions.

#### l-THP food reward control

Food reward studies were conducted in behavioral chambers (Med Associates Inc, St. Albans, VT) on a separate group of rats. Rats were again food restricted to maintain their present weight. Rats were trained to nose poke under an FR 10 schedule for a delivery of sucrose pellets (Bioserv, Flemington, NJ). Sessions timed out after 1-h, 20-s time outs occurred after each pellet delivery. Once animals received the maximum number of pellets in the session (40 pellets) for three consecutive sessions, *l-*THP testing began. Rats were pretreated with saline, 3, 5, 7, or 9 mg/kg *l-*THP (i.p.) 30 min prior to food reinforcement training.

### Microdialysis Experiments

#### Microdialysis surgery

Microdialysis surgeries were performed as described previously [29] in a separate group of rats. Briefly, rats were anesthetized under Equithesin (20 mg/kg) and probes made of 22 g 1/2 needles with 8 mm silica, and 2 mm exposed membrane were implanted into the shell of the nucleus accumbens (A +2 mm, L +1 mm from bregma, V-8 mm to dura). Rats were placed in individual hemispheric bowls to recuperate. Experiments were performed the following day to minimize surgery-induced neurotransmitter release.

#### Microdialysis procedure

Microdialysis experiments were performed in the same hemispheric bowls in which rats stayed overnight. Rats were connected to pumps (Bioanalytical Systems Inc, W. Lafavette, IN) with ringer solution (sodium chloride: calcium chloride: potassium chloride, filtered with 25 mm 0.2um syringe filter) infused at a flow rate of 1 ul/min. DA 10^−8^M was used as an external standard and was tested at least twice immediately before baseline measures were taken to ensure 10 % or less variability in the system (peak of the standard concentration was 100 fmol/min). Baseline DA measures were taken every 20 min until three consecutive samples displayed variability of no more than 17 %. Once this criterion was met, rats were given 5 mg/kg *l-*THP (i.p.) or 0.4 mg/kg nicotine (s.c.) (control groups); or pretreated with 5 mg/kg *l-*THP, 40 mg/kg bupropion (i.p.) (40 min), or 1 mg/kg varenicline (i.p.) (2-h) then given 0.4 mg/kg nicotine (s.c.). Dialysis samples were taken every 20 min over 3-h post nicotine injection. Samples were injected without extraction or purification into Dionex UltiMate 3000 HPLC (Chelmsford, MA) coupled to ESA Coulochem III electrochemical detector (Chelmsford, MA), monosodiumphosphate buffer in methanol/water (10:74:16, v/v/v).

### Histology

At the end of microdialysis experiments, rats were overdosed with phenobarbital. Probes were removed and brains fixed in 20 % formalin for at least two weeks. Brains were cut by vibratome 1000 plus (The vibratome company, St. Louis, MO) into serial coronal slices of 1 cm thickness to verify the placement of probes. Indentation marks left from probes were observed to ensure probe placement was within the nucleus accumbens.

### Statistics

Statistical analysis was carried out by GraphPad 5.00 for windows (La Jolla, CA). Student’s *t*-test, one-way or two-way ANOVA was used to assess significance according to experimental design. When appropriate one or two-way ANOVA was followed by Bonferroni *post hoc* test of significance. Significance was reported at *p <* 0.05 or lower. All analysis was performed on the raw data. For data presentation, a percent of control measure was used to display the observed effects of compounds on SA and reinstatement testing. The percent of control was calculated by taking the three day mean of each individual rat within the same treatment group and dividing it by the three day mean of the entire treatment group during nicotine baseline. In SA testing the test average is presented as a percentage of nicotine baseline response. In reinstatement testing extinction and reinstatement are displayed as a percentage of nicotine baseline response.

## Results

### Effect of l-THP on nicotine SA

**Fig. 1:**
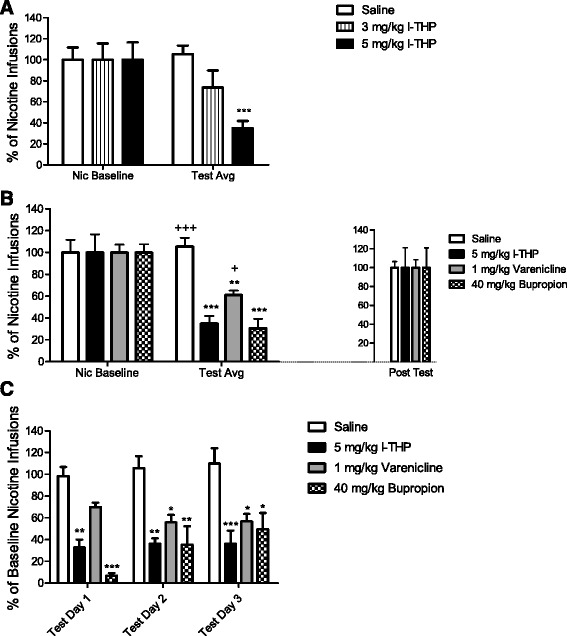
Effect of *l-*THP on nicotine SA and comparison to varenicline and bupropion. *l-*THP decreased nicotine SA in animals trained to stably respond for nicotine infusions. Each pretreatment was tested for three consecutive days all data is presented as mean + S.E.M. **a** Infusions per session over five days of training (Nic Baseline) and three days of treatment (Test Avg) respectively. Saline demonstrated no effect on nicotine SA, while *l-*THP at 5 mg/kg displayed a significant effect on nicotine responding. ***p < 0.001, *t*-test between test avg and nic baseline, *n* = 6 per group, 3 mg/kg *l-*THP group *n* = 12. **b** Infusions per session averaged in the same manner as the previous panel. *l-*THP, varenicline, and bupropion significantly reduced nicotine responding compared to respective nicotine baselines, two-way ANOVA **p < 0.01, ***p < 0.001. + denotes comparison of test averages to 5 mg/kg *l-*THP; the test average of 5 mg/kg *l-*THP was significantly lower than that of varenicline and saline, +*p <* 0.05, +++*p <* 0.001, *n* = 6 per group. All groups rebounded to baseline responding for nicotine in post test sessions. **c** Session breakdown of pretreatment across three day repeated testing of nicotine SA. 5 mg/kg *l-*THP and 1 mg/kg varenicline performed stably across the three day test period while 40 mg/kg bupropion did not, two-way ANOVA with Bonferroni *post hoc* analysis, all treatment groups were compared to saline treatment, *p < 0.05, **p < 0.01, ***p < 0.001

To assess the efficacy of *l-*THP in treatment of nicotine addiction, naive rats were trained to self-administer nicotine under an FR of 5. Rats were treated with saline (1 ml/kg i.p.) or *l-*THP (3 mg/kg or 5 mg/kg, i.p.) before placement into SA chambers. Both doses of *l-*THP displayed a trend of lowering the amount of nicotine infusions taken per session; however, only 5 mg/kg *l-*THP was statistically significant. Figure 1a and Additional file 1: Figure S1a) displays the effects of *l-*THP on nicotine SA over the testing period. Two-way ANOVA for repeated measurements was used to analyze the data with defining factors of pretreatment (saline v. 3 mg/kg *l-*THP v. 5 mg/kg *l-*THP) and session (nic baseline v. test avg). There was a significant effect of session [F (1, 21) = 6.871, *p <* 0.05]. The test average of 5 mg/kg *l-*THP was significantly reduced in comparison to its nicotine average (*t*-test, *p <*0.001).

### Effects of varenicline and bupropion on nicotine SA and comparison with l-THP

A separate group of rats were treated with varenicline (1 mg/kg) or bupropion (40 mg/kg) before placement into SA chambers. Figure 1b and Additional file 1: Figure S1b) displays that like *l-*THP, these treatments significantly reduced nicotine SA. Two-way ANOVA for repeated measures was used with the defining factors of pretreatment (saline v. 5 mg/kg *l-*THP v. 1 mg/kg varenicline v. 40 mg/kg bupropion) and session (nic baseline v. test avg). A significant effect of interaction between pretreatment and session F(3,20) = 6.725, *p <* 0.01; and session F(1,20) = 38.39, *p <* 0.001, was observed. Bonferroni *post hoc* analysis revealed a significant effect of 5 mg/kg *l-*THP (*p <* 0.001), 1 mg/kg varenicline (*p <* 0.05) and 40 mg/kg bupropion (*p <* 0.001) compared to saline treatment. Student’s t-test analysis revealed that *l-*THP reduced nicotine infusions significantly greater than varenicline (*p <*0.05) and equal to bupropion. In a breakdown of testing over the three day period (Fig. 1c and Additional file 1: Figure S1c), two-way ANOVA revealed a significant effect of pretreatment, F(3,40) = 11.89, *p <* 0.001. In Bonferroni *post hoc* analysis, *l-*THP significantly reduced the number of nicotine infusions taken each day (*p <* 0.01 days 1 and 2, *p <* 0.001 day 3). Varenicline significantly reduced nicotine infusions on days 2 and 3 (*p <* 0.05). Bupropion, displayed varying significance in blocking nicotine infusions across test days (*p <* 0.001 day 1, *p <* 0.01 day 2, *p <* 0.05 day 3).

### Effect of l-THP on combined cue and nicotine-induced reinstatement of drug-seeking

**Fig. 2:**
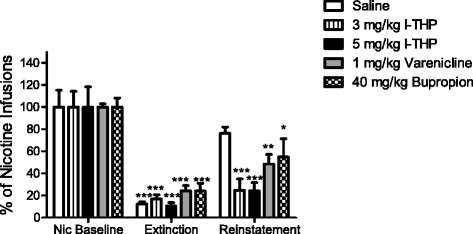
Comparison of pretreatments on nicotine reinstatement. All groups extinguished nicotine-seeking behavior when environmental cues as well as nicotine were removed. Reinstatement consisted of pretreatment followed by a nicotine priming injection 5 min prior to the session as well as reintroduction of the environmental cues associated with nicotine infusions. Nicotine was not available during reinstatement sessions. All drug based pretreatments significantly reduced reinstatement responding from reaching nicotine baseline levels (Nic Baseline). All data is presented as mean + S.E.M. comparisons are made against Nic Baseline within group, two-way ANOVA, *n* = 6 per group, *p < 0.05, **p < 0.01, ***p < 0.001

At the completion of SA testing, extinction was imposed. Rats responded at low levels, below 25 % of nicotine baseline responding within 10 sessions. Reinstatement testing was used to illustrate the effect of *l-*THP and other pretreatments on the prevention of relapse to nicotine-seeking behavior. Figure 2 and Additional file 1: Figure S2 illustrates that pretreatment with both doses of *l-*THP significantly blocked reinstatement to previous nicotine-seeking behavior while pretreatment with saline did not, a significant effect of interaction between session (baseline v. extinction v. reinstatement) and pretreatment F(8, 50) = 7.075, *p <* 0.0001 and session was revealed, F(2,50) = 128.4, *p <* 0.0001.

### Effects of varenicline and bupropion on combined cue and nicotine-induced reinstatement and comparison with l-THP

Rats treated with varenicline and bupropion underwent extinction in the same manner. When tested under reinstatement conditions, both drug treatments significantly blocked reinstatement to previous nicotine-seeking behavior. Bonferroni *post hoc* analysis, from the reported two-way ANOVA analysis on reinstatement testing, revealed significant differences in nicotine baseline (Nic Baseline) and reinstatement for varenicline and bupropion, *p <* 0.01 and *p <* 0.05 respectively. In comparison to bupropion and varenicline, *l-*THP pretreatment, both 3 mg/kg and 5 mg/kg performed superiorly, as seen in Fig. 2. Bonferroni *post hoc* analysis revealed a significant difference in nicotine baseline and reinstatement for both *l-*THP doses, *p <* 0.001.

### Effect of l-THP on nicotine-induced hyperactivity

**Fig. 3:**
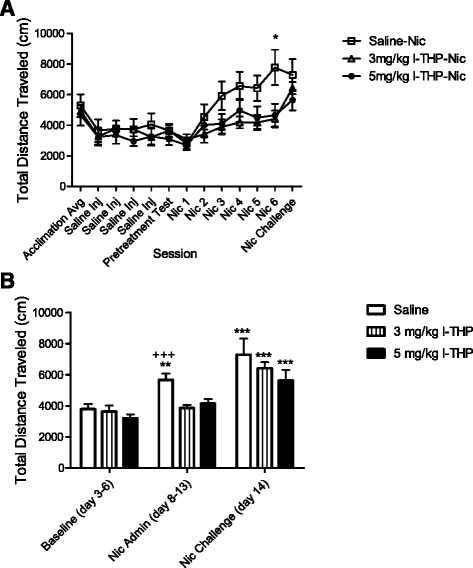
*l-*THP effect on nicotine-induced hyperactivity. *l-*THP displayed a protective effect against nicotine induced hyperactivity. In testing of hyperactivity, all rats were pretreated with two injections of saline during the baseline phase. In the nicotine administration phase (Nic Admin) rats were pretreated with their respective treatment before placement into chambers and treated with nicotine 5 min before placement into chambers. **a** Total movements displayed over each session. All groups display similar movements from acclimation through pretreatment test. When groups are given their respective pretreatments followed by nicotine, differences emerge between groups, significance is seen individually on day 13 (Nic 6) *p < 0.05. **b** Hyperactivity testing collapsed by segment: Baseline, Nic Admin, or Nic Challenge. During the nicotine administration phase rats pretreated with *l-*THP did not display hyperactivity compared to their respective baseline activity. Rats pretreated with saline did display hyperactivity compared to their baseline. During the nicotine challenge (Nic Challenge) all rats received only one injection of nicotine (0.4 mg/kg s.c.) 5 min before placement into locomotor chambers. All rats displayed hyperactivity in this phase. Rats previously pretreated with *l-*THP who did not display hyperactivity during the nicotine administration phase rebounded to display hyperactivity when *l-*THP was not presented before nicotine. All data is presented as mean + S.E.M. Two-way ANOVA * denotes within group comparison to baseline, **p < 0.01, ***p < 0.001, + denotes between group comparison of *l-*THP and saline during Nic Admin, student paired t-test, +++ *p <* 0.001, *n* = 8 per group

The psychostimulant effects of nicotine can manifest in the form of locomotor hyperactivity. Locomotor activity testing was conducted to assess the effect of l-THP on nicotine induced hyperactivity. *l-*THP significantly reduced nicotine-induced hyperactivity when administered as a pretreatment in the nicotine administration phase as shown in Fig. 3a and b, two-way ANOVA revealed an effect of pretreatment [F (2, 231) = 5.412, *p <* 0.01, *n* = 24] and session (baseline v. nicotine administration v. nicotine challenge) [F (2,231) = 17.22, *p <* 0.0001, *n* = 24]. Saline did not block nicotine-induced hyperactivity. Bonferroni *post hoc* analysis revealed significant differences between *l-*THP and saline treated animals (*p <* 0.001) during nicotine administration. When *l-*THP was removed on test day, previous *l-*THP treated animals that displayed no hyperactivity, rebounded to display hyperactivity comparable with saline treated animals (*t*-test analysis of *l-*THP baseline and *l-*THP test-day yielded a significance of *p <* 0.001).

### Effect of l-THP on locomotor activity

**Fig. 4:**
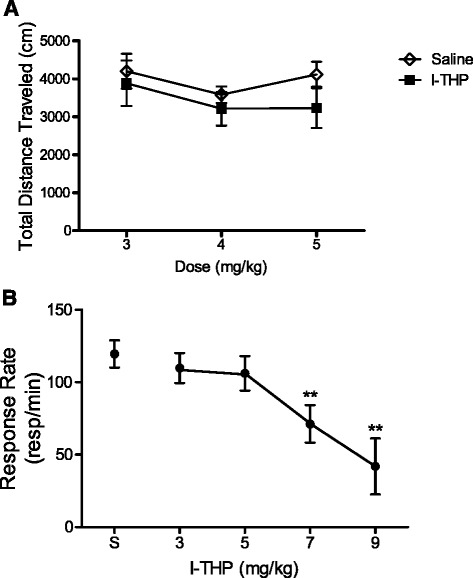
Locomotor and food behavioral control studies of *l-*THP. *l-*THP 3-5 mg/kg displayed no sedative effects on locomotor activity or responding for food, all data is presented as mean + S.E.M. **a** In locomotor testing, rats were acclimated to locomotor chambers. Rats were then pretreated with saline or *l-*THP 30 min before placement into locomotor chambers and treated with saline 5 min before placement into locomotor chambers. Rats were trained under this protocol for six consecutive days. No significant difference was found in locomotor activity between saline and *l-*THP between the doses of 3–5 mg/kg, *n* = 8 per group. **b** In food testing, rats were trained to respond in operant chambers under an FR 10, 20-s time out schedule for sucrose pellets. Sessions lasted 1-h, once rats received the maximum number of pellets (40 pellets) for three consecutive sessions they were tested with pretreatment of *l-*THP in escalating doses; at least five sessions of saline treatment were given between test doses. *l-*THP at 7 mg/kg and 9 mg/kg significantly reduced the responding rate for sucrose pellets within the 1-h session, one-way ANOVA, ** *p <* 0.01, *n* = 10 per group

Given the potential for *l-*THP to induce inhibitory effects, it was crucial to ensure that the therapeutic doses of *l-*THP concerning nicotine addiction did not create sedative effects. To this end, the effect of *l-*THP alone on locomotor activity was tested. Figure 4a displays that *l-*THP alone in the therapeutic range of 3–5 mg/kg did not affect locomotor activity. No significant difference was found between the mean distance traveled by animals pretreated with *l-*THP or saline (all *p’s > *0.14). See Additional file 1: Figure S3 for comparison of l-THP vehicle to saline.

### Effect of l-THP on food-maintained behavior

To further ensure sedative effects of *l-*THP were not observed within our therapeutic range, we evaluated the effect of *l-*THP on operant responding for food. Figure 4b displays that *l-*THP in the therapeutic range of 3–5 mg/kg did not produce inhibitory effects on natural reward (food-sucrose pellets). No significant difference in responding for pellets compared to saline pretreatment was found in this treatment range. Doses greater than 5 mg/kg significantly reduced the rate of responding for sucrose pellets one-way ANOVA [F(4, 36) = 2.909, *p* < 0.01]. These results further validate that behavioral changes observed with 5 mg/kg *l-*THP pretreatment were not a result of disruptive effects of *l-*THP on operant behavior in general.

### Effects of l-THP, varenicline, and bupropion on extracellular DA levels in the nucleus accumbens shell

**Fig. 5:**
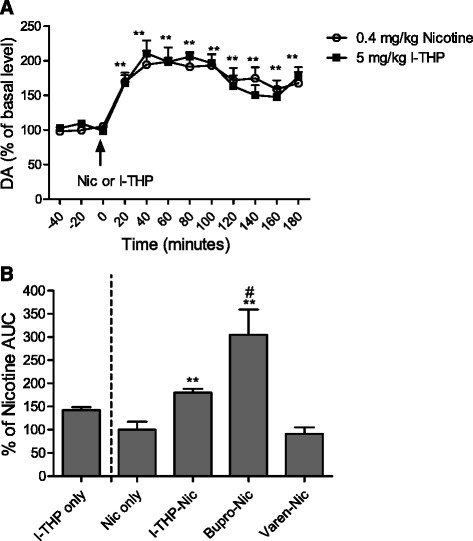
Microdialysis studies of treatment effects on DA release in nAcb. All compounds increased extracellular DA concentrations from baseline within the nAcb. Microdialysis was performed in awake, freely moving rats. Baseline measures were taken in each rat until three consecutive basal DA samples displayed less than 17 % variance. Rats were then pretreated with their respective compounds. Samples were taken every 20 min for 180 min after nicotine administration (or *l-*THP administration when only *l-*THP was given), all data is presented as mean + S.E.M. **a** Effect of nicotine or *l-*THP on extracellular DA concentration in nAcb. Both compounds significantly increased DA concentration from baseline within the nAcb, one-way ANOVA **p < 0.01. **b** Area under the curve (AUC) analysis and comparison of pretreatments to area under the curve of nicotine treated rats (AUC collected from time of pretreatment to 60 min after nicotine was administered). The *l-*THP only group is displayed to show a comparison of changes in extracellular DA concentration caused by *l-*THP under nicotine-free conditions within the same 60 min time frame. *l-*THP pretreated rats who received nicotine and bupropion pretreated rats who received nicotine had significantly higher AUCs than rats receiving nicotine alone. Bupropion pretreated rats receiving nicotine had the highest AUC, one-way ANOVA * denotes comparison to nicotine only group, # denotes comparison to *l-*THP-Nic group, **p < 0.01, # *p <* 0.05, *n* = 4–6 per group

In a separate group of rats, microdialysis probes were implanted in the nAcb (see Additional file 1: Figure S4) to gain an understanding of the neurochemical changes of DA that occur during administration of *l-*THP, varenicline, and bupropion in the presence of nicotine. Baseline DA concentrations were consistent across groups, 29.71 fmol/min (l-THP only), 28.43 fmol/min (l-THP + Nic), 24.86 fmol/min (Nic only), 27.29 fmol/min (Bupropion + Nic), and 27.57 fmol/min (Varenicline + Nic). Nicotine (0.4 mg/kg, s.c.) and *l-*THP (5 mg/kg, i.p.) increased extracellular DA concentration from baseline in the nAcb (Fig. 5a) *p <* 0.01. Area under the curve analysis revealed that all compounds increased extracellular DA concentrations from baseline, with bupropion treatment having the most robust effect (Fig. 5b, see also Additional file 1: Figure S5 and Figure S6). One-way ANOVA revealed an effect of pretreatment F (4, 24) = 8.313, *p <* 0.001, with only bupropion pretreatment displaying significant difference from other groups in Bonferroni *post hoc* analysis (*p <* 0.01 bupropion-nicotine vs. nicotine only, *p <* 0.05 bupropion-nicotine vs. *l-*THP only, *p <* 0.001 bupropion-nicotine vs. varenicline-nicotine). *l-*THP alone did not significantly elevate DA concentrations from DA concentrations measured when nicotine was administered alone. However, *l-*THP administered before nicotine did significantly increase DA concentrations in comparison to DA concentrations measured when nicotine was administered alone, *t*-test *p <* 0.01.

## Discussion

Our study found that *l-*THP blocked the abuse-related behavioral effects of nicotine in several rodent models. *l-*THP decreased nicotine SA in experienced animals, blocked combined cue and nicotine-induced reinstatement of nicotine-seeking behavior, and attenuated nicotine induced hyperactivity. This study also demonstrated neurochemical changes induced by *l-*THP administration in the mesolimbic dopaminergic system. Taken together this study adds knowledge to the current profile of *l-*THP through demonstrating efficacious use of *l-*THP in the treatment of drug addiction beyond heroin, cocaine, and methamphetamine addictions. This study strengthens the case for the development of *l-*THP into a clinical treatment for nicotine addiction. The history of safe clinical use in China along with the preclinical efficacy concerning nicotine addiction displayed in the current study makes *l-*THP a worthy candidate for development into a nicotine cessation treatment.

The rewarding effect of nicotine addiction is difficult to break, as annually only 3 % of smokers quit successfully [1]. Thus there is a drastic need for new efficacious treatments that will enhance smoking cessation. Given that reducing the rewarding value of nicotine can aid in decreasing smoking in repeat users [25], *l-*THP appears promising as a smoking cessation treatment. *l-*THP diminished the rewarding effects of nicotine in a dose dependent manner, by reducing SA of nicotine in experienced animals. The efficacy of *l-*THP is also comparable to first line treatments for smoking cessation at their optimum treatment doses and pretreatment times. The dose of varenicline was chosen based on literature which states a low dose (1 mg/kg) of varenicline with long pretreatment time (2-h) most effectively reduces animal responding for nicotine [15]. The dose of bupropion was chosen based on literature which states that a high dose of bupropion (40 mg/kg) with a moderate pretreatment time (30 min) is most effective in reducing animal responding for nicotine [18]. The efficacy of *l*-THP in reducing nicotine SA proved to be significantly greater than the first line smoking cessation treatment varenicline. Further, the consistency of *l-*THP pretreatment on nicotine SA proved to be more stable than bupropion as l-THP consistently decreased responding for nicotine across each day of study, while the effect of bupropion varied across each day of study. *l-*THP developed no tolerant effect on nicotine SA observed over three repeated administrations. These data suggest that *l-*THP may stably reduce smoking in habitual smokers to a greater degree than treatments that are currently available.

The efficacy of *l-*THP extends beyond primary reward as assessed in nicotine SA studies. *l-*THP has been shown to block or reduce the reinstatement of drug-seeking behavior induced by cocaine, heroin, or methamphetamine [8, 19, 28, 34]. This utility of *l-*THP holds true concerning nicotine addiction. We showed that combined cue and nicotine-induced reinstatement was blocked by *l-*THP at both tested doses. The efficacy of *l-*THP was greater than the efficacy of both varenicline and bupropion. Favorable reinstatement data suggest that *l-*THP may be efficacious in the prevention of nicotine relapse in the clinical setting. Given that relapse is a major obstacle to remaining nicotine free, as 80 % of smokers who attempt to quit without assistance return to smoking within a month [1], the ability of *l-*THP to thwart combined cue and nicotine induced reinstatement is a much needed quality in a smoking cessation treatment.

Beyond SA and reinstatement, *l-*THP displayed utility in attenuating behavioral effects associated with nicotine sensitization. Hyperactivity can result from the psychostimulant effects of nicotine. *l-*THP, however, was able to attenuate this effect, blocking hyperactivity from occurring when administered before nicotine. This effect of *l-*THP displays its utility in treating behavioral components of nicotine sensitization. Thus, *l-*THP is effective against the centrally-mediated effects of nicotine which manifest as drug-seeking behavior and hyperactivity. Clinical studies are needed for validation of *l*-THP effects on both drug seeking and hyperactivity in humans. We believe *l-*THP may also reduce anxiety in the clinical setting given that *l-*THP has been found to alleviate anxious behavior as tested via elevated plus maze and open-field testing [16] in preclinical models. If translated into the clinical setting, reduced anxiety may mean reduced nicotine use and relapse, as smokers are noted to return to smoking in order to reduce anxiety [1, 11].

Our current study dose of *l-*THP is selective to the abuse-related effects of nicotine. *l-*THP, at doses that blocked nicotine’s effect, did not affect food-maintained behavior and had no sedative effect on locomotor activity. Furthermore, our maximally effective dose of *l-*THP (5 mg/kg) is below the reported doses of *l-*THP used for blocking effects on other drugs of abuse. For example, *l-*THP has been used for treatment of cocaine addiction at doses up to 20 mg/kg [19, 31] and methamphetamine addiction at doses up to 10 mg/kg [28] in pre-clinical testing without detriment to animal health. This comparison demonstrates that *l-*THP is safely tolerated at the doses used in the current study. Clinical testing is needed to determine the ideal dosing parameters in humans.

Microdialysis studies were not meant to correlate onto behavioral studies, but to independently examine and compare neurochemical changes as a result of *l-*THP administration, nicotine administration, or combined administration of treatments and nicotine. In microdialysis studies both *l-*THP and nicotine independently increased extracellular DA concentration (in comparison to baseline) in the nAcb. The combined administration of *l-*THP and nicotine also increased extracellular DA concentration. This increase was slightly greater than the dopamine increase induced by the combined administration of varenicline and nicotine, yet modest in comparison to the increase induced with the combined administration of bupropion and nicotine.

The finding of *l*-THP enhanced extracellular DA concentrations in microdialysis experiments is consistent with previous literature [21, 31]. However these findings seem counterintuitive given that *l-*THP is a DA receptor antagonist and is not addictive; it is difficult to say with certainty why these effects occur. We, however, believe the antagonism of *l-*THP at multiple DA receptors, including D_1_, D_2_ and D_3_ play a vital role. *l-*THP’s effect on extracellular DA concentration is likely due to its binding to D_2_ autoreceptors. It has been reported that *l-*THP increases cocaine-enhanced DA within the nAcb in a similar manner to D_2_ or D_3_ antagonists [26, 31, 32], suggesting *l-*THP’s activity at D_2_ autoreceptors may incite the release of extracellular DA within the nAcb. Blockade of autoreceptors on the presynaptic neuron would result in disinhibition and the continued release of DA from the presynaptic neuron resulting in accumulation of DA within the synapse. This accumulation of DA could explain the increased extracellular DA concentrations measured in microdialysis. Clinically, the overall increase in extracellular DA concentration within the nucleus accumbens as a result of *l-*THP administration may contribute to alleviation of the hypodopaminergic state that is associated with withdrawal symptoms [1, 3, 10, 14]. This may prove to be a mechanism through which *l-*THP is efficacious in the treatment of drug addiction. Further investigation with *l-*THP is needed to verify this potential effect. Concerning the anti-addiction effects of *l-*THP, it is suggested that the binding of *l-*THP to multiple DA receptor subtypes, as well as its activity at other monoamine receptors may drive its therapeutic effects while inhibiting side effects [31]. The broad activity of *l-*THP upon binding multiple receptors allows for the modification of various monoamine systems. Specifically, secondary actions at 5-HT and alpha adrenergic receptors may decrease extrapyramidal effects associated with traditional DA receptor antagonists [30]. Thus, *l-*THP has the unique potential to be developed into a treatment with dual capacity, reducing the rewarding pleasure of nicotine and attenuating withdrawal from nicotine with minimal side effects.

Despite the results of the current study, the exact mechanism of action for *l-*THP is still widely unknown. Unlike traditional DA antagonists used pharmacologically, the affinity of *l-*THP to DA receptors is modest, displaying k_i_ values of 124 nm, 388 nm, and 1420 nm for D_1_, D_2_, and D_3_ receptors respectively [30]. Further, the preferential binding of *l-*THP to D_1_ over D_2_, and D_3_ receptors separates it from previously studied DA antagonists (ex. Haloperidol), thus *l-*THP and its pharmacological effects are unlike previously studied compounds.

## Conclusions

This study demonstrates the efficacy and unique neurochemistry of *l-*THP concerning nicotine addiction. This study does not elucidate the exact mechanism underlying the efficacy of *l-*THP in the treatment of nicotine models of addiction; however, it provides evidence for the continued development of *l-*THP into a treatment for nicotine addiction. Future studies may involve testing of *l*-THP preclinically on varying doses of nicotine as well as progressive ratio self-administration experiments. Future clinical studies with nicotine are also in need for further development of *l-*THP into a treatment for nicotine addiction.

## Additional file

Additional file 1: Figures S1-S6. Additional files include the raw self-administration and reinstatement data prior to the percent of control conversion which was performed for visualizing the data. The data from the raw numbers were used for all analyses. Figures S1-S6 also include preliminary locomotor data comparing vehicle (2 % tween-80, 3 % ethanol, 95 % sterile water) control and saline control, a diagram of probe placement location within the rat brain, and concentration vs. time graphs of microdiaylsis experiments including the significantly elevated DA concentrations observed following bupropion administration.

## Abbreviations

DA: Dopamine
FR: Fixed ratio
*l*-THP: levo-Tetrahydropalmatine
nAcb: Nucleus accumbens
nAChR: Nicotinic acetylcholinergic receptor
NE: Norepinephrine
SA: Self-administration
VTA: Ventral tegmental area
