# Supplementary material for: l-tetrahydropalmatine reduces nicotine self-administration and reinstatement in rats
Source: BMC Pharmacol Toxicol. 2016 Nov 7;17:49. doi: 10.1186/s40360-016-0093-6 (PMC5098281; doi:10.1186/s40360-016-0093-6)
Supplement: Additional file 1: Figures S1-S6. — Additional files include the raw self-administration and reinstatement data prior to the percent of control conversion which was performed for visualizing the data. The data from the raw numbers were used for all analyses. Figures S1-S6 also include preliminary locomotor data comparing vehicle (2 % tween-80, 3 % ethanol, 95 % sterile water) control and saline control, a diagram of probe placement location within the rat brain, and concentration vs. time graphs of microdiaylsis experiments including the significantly elevated DA concentrations observed following bupropion administration. [file 40360_2016_93_MOESM1_ESM.doc]

**Figure S1. Raw data of Nicotine Self administration experiments.**

This is the data displayed in figures 1A-1C in raw form, prior to percent of control conversion. Statistics are the same as raw data was used for all statistical analysis.

**Figure S2. Raw data of nicotine reinstatement experiments.**

This is the data displayed in figure 2 in raw form, prior to percent of control conversion. Statistics are the same as raw data was used for all statistical analysis.

**Figure S3. Vehicle vs. Saline pretreatment**

In locomotor testing, pretreatment with vehicle (2% tween-80, 3% ethanol, 95% sterile water) displayed no significant difference from pretreatment with saline (t-test, *p* =0.228).


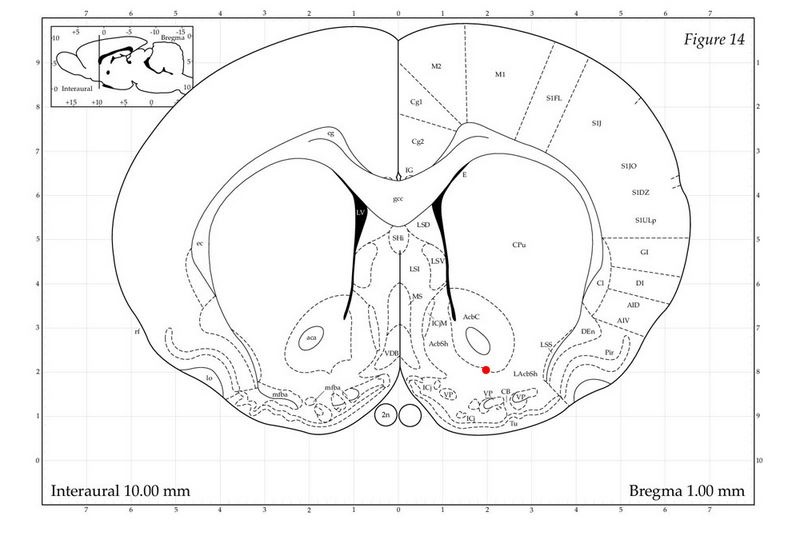


**Figure S4. Microdialysis placement schemata**

Exposed membrane from microdialysis probes were implanted into the shell of the nucleus accumbens (A +2 mm, L +1 mm from bregma, V-8 mm to dura). Probes were cut into 1cm coronal slices via vibratome and probe placement was verified. The red dot symbolizes the target location of probes.

**Figure S5. Effects of various treatments on extracellular DA in nAcb.**

All pharmacological compounds given increased extracellular DA release from basal levels in the nAcb. The increase of DA in the nAcb due to varenicline treatment was the least of all compounds examined.

**Figure S6. Effect of Bupropion on extracellular DA in nAcb.**

Bupropion robustly increased the release of extracellular DA from basal levels in the nAcb. This increase in extracellular DA release was the greatest of all compounds tested. * indicates significant increase in DA release from basal levels. *** p<0.001.
